# Harnessing Microbiome Therapy to Treat Metabolic Syndrome

**DOI:** 10.1016/j.aed.2025.11.015

**Published:** 2026-02-05

**Authors:** Mustafa Al Jnainati, Aishwarya Govindarajan, Srishti Tyagi, Muhammad Iltaf, Jana Al Jnainati, Mohammad Ayoub, Hooria Aiman Shadab

**Affiliations:** 1 University of Bologna, Bologna, Italy; 2 Chettinad Hospital and Research Institute, Kelambakkam, Tamil Nadu, India; 3 Venkateshwara Institute of Medical Sciences, Gajraula, India; 4 Medical Teaching Institution, Ayub Teaching Hospital/Ayub Medical College, Abbottabad, Pakistan; 5 University of Milano-Bicocca, Italy; 6 University of Roma Camillus, Rome, Italy; 7 Ayub Medical College, Abbotabad, Pakistan

**Keywords:** dysbiosis, fecal microbiota transplantation, metabolic syndrome, microbiome, preventive medicine, probiotic

## Abstract

**Background/Objective:**

Metabolic syndrome, a global health crisis marked by insulin resistance, obesity, and dyslipidemia, necessitates novel therapeutic approaches beyond conventional symptom management. Emerging research highlights the gut microbiome as a pivotal modulator of metabolic health, with dysbiosis—characterized by reduced microbial diversity and proinflammatory shifts—implicated in disease pathogenesis. This review synthesizes evidence from preclinical and clinical studies on microbiome-targeted therapies, including fecal microbiota transplantation, designer probiotics, and synbiotics, which aim to restore microbial balance and ameliorate metabolic dysfunction.

**Case Report:**

This review synthesizes evidence from preclinical and clinical studies on microbiome-targeted therapies, including fecal microbiota transplantation, designer probiotics, and synbiotics, which aim to restore microbial balance and ameliorate metabolic dysfunction.

**Discussion:**

Fecal microbiota transplantation transfers beneficial microbiota to enhance insulin sensitivity, while probiotics and synbiotics modulate inflammation, strengthen gut barrier integrity, and stimulate metabolic regulators like glucagon-like peptide-1 and short-chain fatty acids. Mechanistically, these therapies mitigate systemic inflammation, improve glucose/lipid homeostasis, and reduce intestinal permeability linked to endotoxin translocation. Clinical trials report improved glycemic control, lipid profiles, and weight management, underscoring their multitargeted potential. However, challenges such as donor variability, lack of standardized protocols, and long-term safety concerns hinder widespread application. Personalized approaches, informed by machine learning and microbial biomarkers, alongside innovations in Clustered Regularly Interspaced Short Palindromic Repeats-based engineering and encapsulation technologies, may address these limitations.

**Conclusion:**

Despite promising outcomes, rigorous large-scale trials and interdisciplinary collaboration are essential to validate efficacy, optimize delivery, and ensure ethical compliance. In conclusion, microbiome therapies represent a paradigm shift in treating metabolic syndrome by targeting root causes, yet translating preclinical success into clinical practice demands further innovation and evidence-based standardization.

**Highlights**

• Gut dysbiosis drives metabolic syndrome, but fecal microbiota transplantation (FMT), designer probiotics, and synbiotics re-establish microbial diversity, strengthen gut barrier, and activate glucagon-like peptide-1, peptide YY, short-chain fatty acid and bile-acid signaling to restore metabolic homeostasis
• Across 100 studies (2010-2024), lean-donor FMT increased insulin sensitivity by ˜ 25% and lowered hemoglobin A1c 0.6%, while engineered *Lactobacillus/Bifidobacterium* strains improved LDL-C and reduced visceral fat in pre-clinical and early-phase trials
• Network comparisons show FMT most effective for glucose control, synbiotics superior for lowering CRP and triglycerides, and lifestyle therapy remains cost-effective for weight reduction—informing personalized intervention choice
• Next-generation tools—CRISPR/dCas9 microbial editing, multilayer encapsulation, and machine-learning biomarker panels—promise safer, precision-targeted microbiome modulation with real-time response monitoring

**Clinical Relevance**

Targeting the gut microbiome offers a disease-modifying strategy for metabolic syndrome. By restoring microbial balance and triggering endocrine pathways that govern insulin sensitivity, lipid metabolism and inflammation, microbiome therapies can complement or surpass conventional drugs, providing personalized, multitargeted control of obesity, diabetes, and dyslipidemia.

## Introduction

The World Health Organization defines metabolic syndrome as a cluster of metabolic abnormalities anchored by insulin resistance, which must be present alongside at least 2 additional risk factors, including hypertension, dyslipidemia, central obesity, or microalbuminuria.^1^ Treatment options include not only drugs but also exercise and a healthy diet, but unfortunately, in today's world, patients often struggle to adhere to these regimens and therefore suffer from lifelong complications.^2^ Treatment with medication offers symptomatic relief only and isn’t curative; therefore, research on gut microbiome focusing on new sustainable options has begun to show promise.^3^

Our digestive system comprises trillions of microorganisms like bacteria, viruses, and fungi, which play a part in all the physiologic functions from digestion to immunity.^4^ The gut microbiome breaks down food, regulates inflammation, and produces essential nutrients, producing short-chain fatty acids as its function, through which it helps metabolism, reduces inflammation, and supports insulin sensitivity.^5^ Dysbiosis is when the gut microbiome becomes imbalanced, leading to obesity and diabetes. We know through research that people with metabolic syndrome have reduced microbial diversity and an increase in proinflammatory bacteria, worsening insulin resistance and inflammation.^6^ Therefore, the gut microbiome is a determinant of metabolic syndrome. Fecal microbiota transplantation is when healthy gut bacteria from a donor are transferred to a patient to reset the microbiome. Studies have suggested that FMT can improve insulin sensitivity and reduce inflammation.^7^ Designer probiotics are another new approach where engineered bacteria are designed to perform specific bacterial functions in the gut.^8^ The difference between regular probiotics and these is that they are targeted to reduce inflammation, providing general gut health benefits. This increasing interest in studying the gut microbiome is because it helps deal with multiple metabolic risk factors in one shot. It focuses on treating the root issue rather than just the symptoms.^9^

This paper aims to explore the therapeutic potential of microbiome-based interventions, such as probiotics, prebiotics, and fecal microbiota transplantation, in the management of metabolic syndrome, with a focus on their mechanisms of action, current clinical evidence, and future applicability.

## Methodology

Papers published between January 2010 and December 2024 were analyzed across databases such as Google Scholar and PubMed using keywords such as microbiome, metabolic syndrome, probiotic, fecal microbiota transplantation, dysbiosis, and 100 papers were selected for this literature review.

## Discussion

### Mechanistic Links Between the Gut Microbiota and Metabolic Syndrome

Metabolic syndrome is a cluster of conditions that include obesity, insulin resistance, and high blood pressure, leading to an imbalanced gut microbiome. Fecal microbiota transplant and engineered probiotics are used to treat microbial imbalance.^10^ Bacteria in the gut break down dietary fibers into short-chain fatty acids (SCFAs) like acetate and butyrate. They become messengers, boosting insulin sensitivity, reducing inflammation, and strengthening the gut lining to prevent leakage of harmful substances.^11^^,^^12^ They also signal the brain for satiety and help maintain weight. Gut microbes help digest fat by processing bile acids, and activate receptors that stabilize blood sugar and cholesterol. If it is imbalanced, it can worsen metabolic issues for which FMT is used to restore healthy bile acid metabolism.^13^ A disrupted gut microbiome (dysbiosis) can contribute to increased intestinal permeability, allowing bacterial endotoxins such as lipopolysaccharides (LPS) to translocate into the bloodstream. This process triggers chronic low-grade inflammation, which is implicated in the development of insulin resistance. Akkermansia muciniphila is a probiotic that can patch up the lining of the gut, whereas SCFAs from fiber-fermenting bacteria calm inflammation by blocking pro-inflammatory molecules.^14^ The gut and brain are in constant contact. Hormones like glucagon-like peptide-1 and peptide YY (PYY), which control hunger and blood sugar, are produced through gut bacteria. In case of disruption, designer probiotics help rewire the communication by boosting glucagon-like peptide-1or FMT.^15^ Figure summarizes the discussed mechanistic links. Genetics is also a factor, some people are born with more natural inflammation-fighting bacteria, and their lifestyle plays a bigger role, wherein a high-fat diet, antibiotics, and stress will destroy the beneficial microbes, leading to metabolic dysfunction. The gut–liver axis integrates dietary, microbial, and hormonal signals to regulate hepatic triglyceride (TG) storage and lipid metabolism. Hepatic lipid load primarily derives from portal free fatty acids from visceral lipolysis, carbohydrate-driven de novo lipogenesis in hepatocytes, and intestinal chylomicron remnants; TG accumulation reflects the balance of these inputs with VLDL export.^16^ Microbial signals tilt this balance: SCFAs support barrier integrity and insulin sensitivity, whereas LPS–TLR4 signalling in hepatocytes and Kupffer cells induces inflammatory cascades and lipogenic transcription (SREBP-1c, ChREBP). Microbiota-modified bile acids signal via FXR/TGR5 and ileal FGF19 to regulate TG synthesis, β-oxidation, and cholesterol handling.^17^^,^^18^ Collectively, these pathways increase TG storage, provoke ER/oxidative stress, and drive lipid-droplet accumulation—linking dysbiosis to steatosis and progression toward NASH within metabolic syndrome. Designer probiotics or fecal microbiota transplantation have been utilized to counteract these effects in individuals at risk of metabolic dysfunction.^19^

**Fig:**
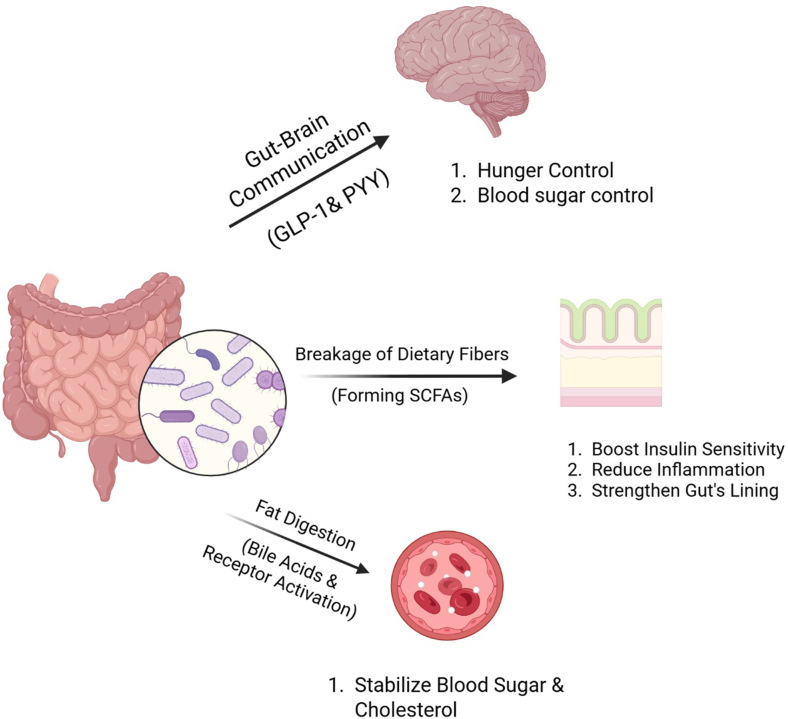
Gut microbiota and nutrient metabolism. *GLP-1*, glucagon-like peptide-1; *PYY*, peptide YY; *SCFAs*, short-chain fatty acids.

### Overview of Gut Microbiome Therapies Relevant to Metabolic Syndrome

Metabolic syndrome is a cluster of conditions that increase the risk of heart disease, stroke, and type 2 diabetes.^20^ Recent research has shown that the gut microbiome plays a crucial role in the development and progression of metabolic syndrome.^21^ Numerous studies have demonstrated the link between gut microbiota dysbiosis and metabolic syndrome.^22^ Imbalances in the gut microbiome have been associated with obesity, insulin resistance, and inflammation, which are key components of metabolic syndrome.^23^^,^^24^ Various gut microbiome therapies are:

FMT is a medical procedure that transfers fecal matter from a healthy donor into a patient's gastrointestinal tract to restore a balanced gut microbiome.^25^ FMT aims to introduce beneficial microorganisms into the gut to treat various gastrointestinal diseases, including recurrent Clostridium difficile infection, inflammatory bowel disease, irritable bowel syndrome (IBS), etc.^25^, ^26^, ^27^, ^28^, ^29^ Beyond them, FMT is being explored for the treatment of non-GI disorders, including metabolic syndrome. It has been shown to improve insulin sensitivity and increase butyrate-producing intestinal microbiota in patients with metabolic syndrome.^30^ Animal and small-scale human studies have demonstrated the potential of FMT in treating metabolic syndrome by improving insulin sensitivity, glucose metabolism, and lipid profiles.^27^^,^^31^^,^^32^

Probiotics are live microorganisms that provide health benefits when administered in adequate amounts. They can be categorized into 2 types: traditional and designer probiotics. Traditional probiotics are naturally found in fermented foods, such as yogurt and sauerkraut, and include strains like Lactobacillus acidophilus and Bifidobacterium bifidum.^33^^,^^34^^,^^35^ Designer probiotics, on the other hand, are genetically engineered or specifically selected microorganisms designed to provide specific health benefits, such as Lactobacillus plantarum 299v and Bifidobacterium lactis BB-12.^36^^,^^37^

Probiotics have been shown to provide various health benefits, including improving digestive health, boosting the immune system, reducing symptoms of IBS, improving mental health, and supporting weight management.^38^ They exert their beneficial effects through several mechanisms of action, including modulating the gut microbiota composition and activity, interacting with the host immune system, and producing bioactive compounds.^33^^,^^37^^,^^35^^,^^39^ They adhere to intestinal epithelial cells, forming a barrier against pathogenic bacteria and reducing infection and inflammation risk.^38^ Designer probiotics can be engineered to express specific proteins or peptides, interacting with host cells to exert beneficial effects. Emerging designer probiotics producing anti-inflammatory cytokines, antioxidants, neurotransmitters, and antimicrobial peptides have shown promise in reducing inflammation, oxidative stress, and improving cognitive function.^40^, ^41^, ^42^, ^43^

Prebiotics are nondigestible fibers and oligosaccharides that serve as food for beneficial microorganisms in the gut, promoting their growth and activity.^44^ They help increase the populations of beneficial microbes.^45^ Synbiotics, combinations of prebiotics and probiotics, work together to promote the growth of beneficial microbes. The prebiotic component provides food for the probiotic microorganisms, enhancing their survival, growth, and activity.^44^ Synbiotics have been shown to have synergistic effects and have more benefits than prebiotics.^46^ They can enhance the colonization of beneficial microbes, increase the production of SCFAs, and improve symptoms of IBS and inflammatory bowel disease.^45^, ^46^, ^47^^,^^48^

### Evidence from Clinical and Preclinical Studies

FMT has shown promise in treating metabolic syndrome, with several human trials demonstrating its potential benefits. Studies have reported improvements in insulin sensitivity, glucose metabolism, and lipid profiles in patients with metabolic syndrome.^29^^,^^49^^,^^50^ These findings suggest that FMT may be a useful adjunctive therapy for metabolic syndrome.

Animal model research has provided valuable insights into the mechanisms of FMT. Studies have shown that FMT can improve glucose metabolism and insulin sensitivity by altering the gut microbiota composition and increasing the production of SCFAs.^51^ FMT has also been found to reduce inflammation and improve symptoms in mouse models of colitis and obesity.^52^ For FMT to be successful, several factors are crucial. Donor selection is critical, as the donor's microbiota composition can significantly impact the recipient's response to FMT.^53^ Engraftment, or the successful colonization of the recipient's gut with the transplanted microbiota, is also important.^54^ Long-term efficacy is a concern, but some studies have reported sustained benefits up to several years after FMT.^55^

Probiotic supplementation has positively impacted metabolic parameters, including glucose control, lipid profiles, and obesity. Meta-analyses found that probiotics improved fasting blood glucose levels and hemoglobin A1c (HbA1c) in patients with type 2 diabetes, and reduced body mass index and body weight in obese individuals.^56^^,^^57^ Probiotics have also been found to improve lipid profiles, reduce total cholesterol and triglyceride levels, and increase high-density lipoprotein levels.^41^ Strain-specific effects have been observed, with Lactobacillus acidophilus improving glucose metabolism and reducing inflammation in patients with type 2 diabetes, and Bifidobacterium lactis B-420 reducing body weight and improving lipid profiles in obese individuals.^58^^,^^59^ Dosage and duration of probiotic supplementation are crucial in achieving the desired metabolic effects. Studies found that specific dosages and durations of probiotic supplementation improved insulin sensitivity, reduced HbA1c levels, and reduced body fat mass.^60^^,^^61^ Designer probiotics have also shown promise in modulating metabolic pathways, improving glucose metabolism and insulin sensitivity in mice.^62^^,^^63^

Combining prebiotics and synbiotics has shown potential in enhancing metabolic health. Pairing the prebiotic inulin with the probiotic Lactobacillus plantarum reduced body weight and improved lipid profiles in obese individuals.^64^ Studies have demonstrated that a synbiotic combination of fructooligosaccharide, Bifidobacteria, and Lactobacilli reduces inflammatory markers and improves glucose metabolism in patients with type 2 diabetes and reduces body fat mass and improves metabolic parameters in obese individuals.^65^^,^^66^ Synbiotics have also positively impacted lipid profiles. A meta-analysis found that synbiotic supplementation reduced total cholesterol and triglyceride levels, while increasing high-density lipoprotein cholesterol levels.^67^ The study in Thai obese individuals found that a synbiotic combination of Lactobacillus paracasei, Bifidobacterium longum, Bifidobacterium breve, inulin, and fructooligosaccharide reduced body weight and other obesity related metabolic biomarkers.^68^ The PREMOTE study demonstrated that a prebiotic supplement improved glucose metabolism and reduced inflammation in patients with type 2 diabetes.^65^ Variations in formulations and dosages are also limitations of current clinical trials. A review of 23 clinical trials on synbiotics concluded that modest effects on body weight and waist circumference were seen^67^ Tables 1 and 2.

**Table 1:** Microbial Therapeutics Tested in Metabolic Disease: Strains, Regimens, and Clinical Outcomes

Study (Y)	Intervention	Strain(s)	Endpoint(s)
Andreasen et al, 2010^107^	Probiotic	Lactobacillus acidophilus NCFM	Improved glucose metabolism, reduced inflammation, increased insulin sensitivity
Kim et al, 2013^57^	Probiotic	Lactobacillus rhamnosus GG	Improved insulin sensitivity, reduced adiposity via adiponectin production
Uusitupa et al, 2020^56^	Probiotic	Bifidobacterium animalis subsp. lactis 420	Reduced body weight, improved lipid profile
Takahashi et al, 2016^58^	Probiotic	Bifidobacterium animalis ssp. lactis GCL2505	Decreased visceral fat accumulation
Liu et al, 2022^59^	Designer probiotic (preclinical)	Lactiplantibacillus plantarum Y15	Improved glucose metabolism via NF-κB and insulin signaling modulation
Kondo et al, 2010^60^	Designer Probiotic (preclinical)	Bifidobacterium breve B-3	Antiobesity effects, improved lipid/glucose metabolism
Hosseinifard et al, 2020^30^	Synbiotic	Lactobacillus plantarum + Inulin	Reduced body weight, improved lipid profile in obese individuals
Dewulf et al, 2013; Nunez-Sanchez et al, 2021^61^^,^^63^	Synbiotic	Fructooligosaccharides + Bifidobacterium spp. + Lactobacillus spp.	Reduced inflammatory markers, improved glucose metabolism, reduced body fat mass
Chaiyasut et al, 2021^65^	Synbiotic (RCT, Thai adults)	Lactobacillus paracasei, B. longum, B. breve + Inulin & FOS	Reduced body weight, improved metabolic biomarkers

Abbreviations: FOS = fructooligosaccharides; GCL = strain designation; GG = Gorbach–Goldin; NCFM = North Carolina Food Microbiology (strain designation); RCT = randomized controlled trial.

**Table 2:** Evidence of Microbiome and Lifestyle Interventions that Improve Metabolic Health

Study (Y)	Intervention	Design/*N* (arms)	Result(s) (effect size/95% CI/*P*-value)	Trial arms/Follow-up	Conclusion	Strengths and limitations
Kootte RS et al., Cell Metab 2017^108^	FMT	Randomized RCT; *n* ≈ 38 (allogenic lean donor vs autologous)	Significant insulin sensitivity improvement at 6 wks; not sustained at 18 wks.	Single FMT; follow-up 18 wk	Replicates short-term benefits but effect fades.	**Strengths:** larger than prior FMT, mechanistic subgroup analysis. **Limitations:** modest sample, transient effects.
Vrieze A et al., Gastroenterology 2012^27^	FMT	Randomized RCT; *n* = 18 (allogenic lean donor vs autologous)	Peripheral insulin sensitivity (Rd) increased from 26.2 to 45.3 μmol kg^-1^·min^-1^ at 6 wk in lean-donor recipients (*P* < 0.05).	Small-intestinal infusion; primary observation at 6 wk	Single lean-donor FMT transiently improved insulin sensitivity.	**Strengths:** pioneering proof-of-concept RCT. **Limitations:** very small *n*, short follow-up, no clinical endpoints.
Proença IM et al., Nutr Res 2020^46^	FMT	Systematic review/meta-analysis of 6 RCTs; total *n* = 154	HbA1c MD = −1.69 mmol/L (95% CI −2.88 to −0.56), *P* = 0.003; HDL MD = +0.09 mmol/L (95% CI 0.02–0.15), *P* = 0.008.	Allogenic FMT vs placebo/autologous	Meta-analysis shows modest pooled effects.	**Strengths:** quantitative synthesis. **Limitations:** few trials, heterogeneity, small samples.
Mocanu M et al., Nat Med 2021^109^	FMT	Randomized, double-blind, placebo-controlled; *n* = 70, 4 arms	FMT + low-fermentable fiber showed modest HOMA2-IR improvement (*P* < 0.05).	Oral capsules ± fiber; 6-wk follow-up	Suggests fiber aids engraftment; effects modest.	**Strengths:** novel oral capsule, rigorous design. **Limitations:** short-term, modest effect, exploratory.
Li G et al., J Transl Med 2023^110^	Probiotics	Systematic review & meta-analysis of ∼30 RCTs; *n* ≈ 1827	FBG SMD = −0.331 (95% CI −0.424 to −0.238), *P* < 0.001; HbA1c SMD = −0.421 (95% CI −0.583 to −0.258), *P* < 0.001; HOMA-IR SMD = −0.224 (95% CI −0.342 to −0.105), *P* < 0.001.	Various probiotic supplements; ≤12 wk	Small but consistent glycemic improvements.	**Strengths:** large pooled sample, robust stats. **Limitations:** heterogeneity in strains/duration, modest effect sizes.
Look AHEAD, NEJM 2013^111^	Lifestyle	Multicenter RCT; *n* = 5145 with T2DM; 9.6 y follow-up	Weight loss: 8.6% vs0.7% at 1 y. No significant CV event reduction (HR 0.95, 95% CI 0.83–1.09, *P* = 0.51).	Lifestyle vs education/support	Improved weight & metabolic outcomes, no CV benefit.	**Strengths:** very large, long-term RCT. **Limitations:** no CV benefit despite weight loss.
DPP, NEJM 2002^112^	Lifestyle	Large multicenter RCT; *n* = 3234 (lifestyle vs metformin vs placebo)	Lifestyle ↓T2DM risk by 58% (95% CI 48–66%); incidence: placebo 11.0 vs lifestyle 4.8 per 100 person-y (*P* < 0.001).	Diet + exercise lifestyle vs metformin vs placebo; 2.8 y follow-up	Landmark trial showing durable diabetes prevention.	**Strengths:** very large sample, rigorous design, robust outcomes. **Limitations:** resource-intensive intervention.
PREDIMED, NEJM 2013^113^	Diet	Randomized trial; *n* = 7447 (MedDiet + EVOO, MedDiet + nuts, control)	Primary CV outcome: MedDiet + EVOO HR 0.70 (95% CI 0.54–0.92).	Mediterranean diet ± supplements; 4.8 y follow-up	MedDiet reduced CV events; some diabetes benefit.	**Strengths:** large primary prevention trial, robust outcomes. **Limitations:** diet adherence variability, retraction/re-analysis controversy.

Abbreviations: FBG-SMD = fasting blood glucose—standardized mean difference; FMT = fecal microbiota transplantation; HbA1c = hemoglobin A1c; HDL = high-density lipoprotein; HOMA-IR = homeostatic model assessment of insulin resistance.

Comparative effectiveness studies have investigated FMT, probiotics, and lifestyle interventions in improving metabolic health. A systematic review found FMT more effective than probiotics in improving glucose metabolism and reducing body weight, while another study found probiotics more effective in reducing inflammation and improving lipid profiles.^29^^,^^30^ Lifestyle interventions were also effective, outperforming probiotics in reducing body weight and improving glucose metabolism.^69^ FMT is costly compared to prebiotics and probiotics, and compliance is lower with FMT.^70^, ^71^, ^72^

## Integrative Insights

Metabolic conditions, including obesity, type 2 diabetes mellitus, and dyslipidemia, change gut microbiota composition.^73^ Dysbiosis (imbalance in the microbiota) impacts insulin sensitivity, lipid metabolism, and systemic inflammation.^74^ These therapies work through various mechanisms. The gut microbiota affects hormonal pathways, which are essential for energy homeostasis. Microbial metabolites, including SCFAs, act on endocrine cells of the gastrointestinal tract to release GLP-1 and PYY, which increase insulin secretion.^75^ Whereas probiotic strains, like Lactobacillus and Bifidobacterium, help in appetite regulation, folic acid generation, and energy balance by increasing the secretion of leptin and ghrelin.^76^^,^^77^

When gut microbiota ferments dietary fibers, they synthesize SCFAs, including acetate, propionate, and butyrate, which act as chemical messengers that enhance glucose metabolism, insulin sensitivity, and lipid balance. G-protein-coupled receptors (GPR41 and GPR43) activated by SCFAs enhance glucose uptake and lipid metabolism.^78^ The composition of the gut microbiota influences SCFA production, with specific bacterial species, such as Faecalibacterium prausnitzii and Roseburia, which produce high butyrate, having been linked to better mitochondrial function and increased energy expenditure. Keep in mind that gut microbiota is influenced by host epigenetic profiling by inhibiting the histone deacetylases, also to improve metabolic functions.^76^^,^^79^^,^^50^ Probiotics and prebiotics increase SCFA levels and promote beneficial bacterial growth, thereby providing metabolic benefits.^75^ FMT therapy samples, when taken from healthy donors, increase butyrate production to improve a cell's sensitivity to insulin.^80^

Dysbiosis worsens chronic low-grade inflammation, a characteristic of metabolic disorders, by enhancing intestinal permeability. Bacterial endotoxins, including LPS, infiltrate the bloodstream and stimulate the secretion of proinflammatory cytokines, such as tumor necrosis factor-alpha and interleukin-6, and this further increases intestinal permeability.^74^ However, the regular use of probiotics and prebiotics strengthens the intestinal barrier by modulating tight junction proteins, including zonula occludens-1, occludin, and claudin, therefore avoiding the translocation of toxic substances into circulation.^76^ A study was conducted on murine subjects by introducing probiotics, which evidenced a diminishment in food consumption, insulin resistance, and a decrease in body weight by modulation of gut flora composition and a rise in the hormone GLP-1. This led to reversing obesity and diabetes in the murine population.^77^ SCFAs, particularly butyrate, inhibit NF-κB signaling, decreasing tumor necrosis factor-alpha and interleukin-6 production, which leads to decreased systemic inflammation.^81^ FMT has minimised inflammation by restoring gut microbial diversity.^73^

The relationship between gut microbiome and bile acid is interdependent, as modified bile acids can affect how our body processes nutrients and change the types of bacteria present in the gut, whereas the gut microbiota also modifies bile acid composition by converting primary bile acids into secondary bile acids, which in turn activate receptors, such as the farnesoid X receptor and Takeda G-protein-coupled receptor 5.^80^ When activated, they help regulate the metabolism of bile acids, fats, and carbohydrates.^80^^,^^82^ FMT and probiotic therapies (e.g., Akkermansia muciniphila) improve cholesterol clearance and insulin sensitivity.^73^

### Challenges and Considerations

Despite the several therapeutic uses of microbiome therapies, several challenges limit their applicability.^83^^,^^84^ To date, there is no concrete definition of the complex composition of FMT or standard screening regulations.^83^^,^^84^ We cannot use the same regulations applied to drugs for fecal microbiota, as it is a complex mixture, and determining its potency, validity, or controlling its production is difficult.^84^ Using the term “virtual organ” to describe it is more accurate; hence, the precautions and regulations followed for tissue transplantations should be practiced here as well.^84^ It is crucial to select and rigorously screen donors and adequately prepare patients to minimize the transmission of diseases such as psychiatric disorders, including long-term depression and anxiety, multidrug-resistant organisms, and improve the efficacy of the treatment regimen.^83^^,^^84^

The efficacy of FMT is partly dependent on the donor. Due to the heterogeneity of gut microbiota across the population, there will be differences in clinical responses; one sample could work for one person, one may reject it, and another may have an adverse effect.^84^, ^85^, ^86^^,^^87^ Upon breakdown, dietary fibers stimulate insulin secretion by increasing GLP-1 and PYY levels, thereby regulating glucose levels. This degradation is influenced by gut microbiota, and its heterogeneity can cause differences in response to fiber intake in Type 2 diabetes patients.^85^^,^^88^

The donor selection process is currently based on exclusion criteria since the term of an ideal donor hasn’t been defined yet. Moreover, the limited knowledge of gut microbiome further hinders the selection of an ideal donor for a specific disease.^84^ Administration of probiotics to obese individuals with a Prevotella-dominant microbiome had more favorable outcomes than Bacteroides-dominant ones.^89^ The microbiome is a biodiversity hotspot consisting of different species of bacteria, viruses, fungi, etc, in varying amounts; this alone can impact the clinical usefulness of the sample and affect the consistency and reliability of microbial therapies.^90^ These issues complicate the definition of what constitutes a healthy ideal microbiome.^86^ We can optimize therapeutic outcomes by tailoring treatment strategies that account for individual differences in metabolic phenotype and gut microbiome makeup and opt for patient-centered protocols.^89^^,^^91^ Adopting a multidisciplinary approach to microbial therapies and interlinking experts in nutrition, endocrinology, and microbiology, we can expand our gut microbe knowledge and overcome the issues stemming from its diversity.^91^ As FMT is derived from human donors, its supply is limited.^83^ The follow-up time frame in the clinical trials was insufficient in determining the potential long-term adverse effects, and if an adverse effect was noted, it was difficult to accurately link it to the microbiome therapy.^84^

Variations in the technology used for the analysis of microbiome samples pose challenges. Most researchers amplify rRNA to study the samples, but there are several technical variables and primers.^92^^,^^93^ Since not all the gut microbes have been identified and inter-person variations exist, certain useful organisms may remain unidentified. This leads to differences in outcomes in different research studies. Regulations must be set to standardize analytic methodologies, keeping in mind that factors such as age and diet modify the samples over time.^86^^,^^93^ To evaluate the use of prebiotics, probiotics, and postbiotics to alter the gut microbiome and influence different metabolic disorders, efforts should be made to create randomized, large-scale, high-quality research, and clinical trials.^89^

### Emerging Directions and Future Perspectives

Newer systems have been developed to deliver microbiome therapies. Using bacteriophages has demonstrated great success in regulating the expression of gut microbes;however, due to their weak structure, it’s easily destroyed by harsh environments during storage, delivery, or in the gut before exerting their full effect.^94^^,^^95^ To combat this, we have encapsulation strategies that improve phage stability and provide precise control over delivery dosage. Different pH and enzyme-specific encapsulation materials were created to improve outcomes.^94^^,^^96^ The structure of hydrogels makes them suitable for encapsulation and targeted delivery of engineered bacteria. These engineered bacteria within the capsules can be designed to release therapeutic contents in specific anatomic areas or when special conditions are met, such as higher degrees of inflammation or to fix any defects in the patient’s microbiome, allowing for a targeted approach.^96^ Electronic devices can be integrated into these capsules to enable the collection, measurement, and processing of data.^96^ Concerns over their possible spread beyond the intended area or unfavorable interactions were resolved with the development of genetic kill switches to destroy the bacteria after the goal is achieved and the usage of multiple polymer layers to restrict leakage of contents.^96^^,^^97^ Trials have shown that combining probiotics and phage products comprising specific E. coli-targeting phages was more effective in reducing gastrointestinal inflammation than monotherapy.^94^ Recently, Clustered Regularly Interspaced Short Palindromic Repeats (CRISPR)/dCas9-based systems have become increasingly popular in microbiome studies.^95^^,^^98^^,^^99^ A study proved the noninvasive combination of encapsulation and CRISPR methods was effective in modifying microbiome composition by regulating transcription in a single dose with minimal physiological distortions.^95^

By leveraging biomarkers, we can enhance precision in medical decision-making, optimize therapeutic outcomes, and develop more personalized treatment strategies. A study established the presence of bacteria such as Prevotella copri, Bacteroides stercoris, etc, in the microbiome of individuals wherein lifestyle interventions had minimal impact on the microbiome, whereas 38 species were identified in the microbiome of those individuals who had a good response to lifestyle interventions.^100^ These species could be used as therapeutic biomarkers and to monitor the effectiveness of interventions. Supplementation with those species such as Faecalibacterium prausnitzii, Fusicatenibacter saccharivorans, etc, would yield better outcomes with lifestyle interventions such as diet and exercise in people with metabolic syndrome.^100^

Machine learning and counterfactual analysis were done on human stool metagenomes of obese individuals across several countries.^101^ Certain species, such as Prevotella copri were found to improve glucose homeostasis by increasing GLP-1 levels and reducing hunger, whereas Bacteroides eggerthii, etc, had positive effects on weight loss and metabolism by producing short-chain fatty acids.^101^ Using them in microbiome therapies and pharmacological interventions such as Metformin or GLP-1 agonists can yield a superior treatment outcome for metabolic syndrome.^101^^,^^102^ In contrast, the elimination of obesity-specific bacteria such as Coprococcus comes, etc, can also be tried for obesity management.^101^ Appropriate gut microbiome modification may offer sustained protection against obesity, however, we shouldn’t generalize these findings across all populations due to environmental differences and limited datasets.^101^^,^^102^ Counterfactual analysis in microbiome research is still in its early stages, and the accuracy of machine learning models needs improvement. Extensive geographic scope research studies should be done incorporating strain-level taxonomy to better validate microbial biomarkers as indicators of obesity across diverse populations.^101^

## Conclusion

Metabolic syndrome, a global health challenge characterized by insulin resistance and associated comorbidities, demands innovative therapeutic strategies beyond symptomatic management. This review underscores the pivotal role of the gut microbiome in modulating metabolic health, emphasizing its potential as a therapeutic target. Dysbiosis, marked by reduced microbial diversity and proinflammatory shifts, exacerbates insulin resistance, inflammation, and lipid dysregulation. Emerging therapies, including FMT, designer probiotics, and synbiotics, demonstrate promise in restoring microbial equilibrium, enhancing insulin sensitivity, and attenuating inflammation through mechanisms such as SCFA production, gut barrier reinforcement, and hormonal regulation (e.g., GLP-1 and PYY). Clinical and preclinical studies highlight improvements in glucose metabolism, lipid profiles, and obesity metrics, positioning microbiome modulation as a holistic approach to address root causes rather than isolated symptoms.

However, challenges persist, including donor variability, lack of standardized protocols, and regulatory complexities. The heterogeneity of gut microbiota across populations necessitates personalized strategies, while long-term efficacy and safety remain understudied. Most studies have only included short-term follow-up, usually around 12 to 18 weeks. In FMT therapy for obesity and metabolic syndrome, early improvements within 6 weeks were observed, but the benefits tends to diminish over time, suggesting a weaning effect. Studies show when treatment is stopped abruptly, it often leads to a rapid decline in the treatment benefits.^103^ They also indicates that epigenetic mediators such as SCFAs work mainly through transcriptional regulation of genes instead of permanent alterations in DNA sequences,^104^ therefore the metabolic and immune-related improvements are reversible once therapy is discontinued.^105^ Data on long-term outcomes after stopping treatment are limited, though one study suggests that the effects of FMT persists for up to a year.^106^ Even so, maintenance dosing is necessary to sustain the benefits. Future research should explore how these epigenetic shifts evolve over time and investigate strategies to make favorable changes more stable. Though permanent lifestyle and dietary changes with intake of prebiotics or postbiotics are must for long term benefits. Future advancements in encapsulation technologies, CRISPR-based microbial engineering, and machine learning-driven biomarker identification may enhance precision and scalability. Integrating multidisciplinary expertise and large-scale randomized trials will be critical to validate these interventions and optimize clinical translation.

In conclusion, microbiome-based therapies represent a paradigm shift in metabolic syndrome management, offering sustainable, multitargeted benefits.

## Author Contributions

All authors contributed equally and attest that they meet the ICMJE criteria for authorship and gave final approval for submission.

## Data Availability Statement

Data included in article/supp. material/referenced in article.

## Disclosure

The authors have no conflicts of interest to disclose.
